# Portrait of Ependymoma Recurrence in Children: Biomarkers of Tumor Progression Identified by Dual-Color Microarray-Based Gene Expression Analysis

**DOI:** 10.1371/journal.pone.0012932

**Published:** 2010-09-24

**Authors:** Matthieu Peyre, Frédéric Commo, Carmela Dantas-Barbosa, Felipe Andreiuolo, Stéphanie Puget, Ludovic Lacroix, Françoise Drusch, Véronique Scott, Pascale Varlet, Audrey Mauguen, Philippe Dessen, Vladimir Lazar, Gilles Vassal, Jacques Grill

**Affiliations:** 1 Université Paris-Sud, CNRS UMR 8203 “Vectorology and Anticancer Treatments”, Gustave Roussy Institute, Villejuif, France; 2 CNRS FRE 2939, Bioinformatics Group, Gustave Roussy Institute, Villejuif, France; 3 Department of Neurosurgery, Necker Sick Children's Hospital, Université Paris V Descartes, Paris, France; 4 Translational Research Laboratory, Gustave Roussy Institute, Villejuif, France; 5 Department of Neuropathology, Sainte-Anne Hospital, Paris, France; 6 Department of Biostatistics, Gustave Roussy Institute, Villejuif, France; 7 Functional Genomics Unit, Gustave Roussy Institute, Villejuif, France; 8 Department of Pediatric and Adolescent Oncology, Gustave Roussy Institute, Villejuif, France; Institute of Cancer Research, United Kingdom

## Abstract

**Background:**

Children with ependymoma may experience a relapse in up to 50% of cases depending on the extent of resection. Key biological events associated with recurrence are unknown.

**Methodology/Principal Findings:**

To discover the biology behind the recurrence of ependymomas, we performed CGHarray and a dual-color gene expression microarray analysis of 17 tumors at diagnosis co-hybridized with the corresponding 27 first or subsequent relapses from the same patient. As treatment and location had only limited influence on specific gene expression changes at relapse, we established a common signature for relapse. Eighty-seven genes showed an absolute fold change ≥2 in at least 50% of relapses and were defined as the gene expression signature of ependymoma recurrence. The most frequently upregulated genes are involved in the kinetochore (ASPM, KIF11) or in neural development (CD133, Wnt and Notch pathways). Metallothionein (MT) genes were downregulated in up to 80% of the recurrences. Quantitative PCR for ASPM, KIF11 and MT3 plus immunohistochemistry for ASPM and MT3 confirmed the microarray results. Immunohistochemistry on an independent series of 24 tumor pairs at diagnosis and at relapse confirmed the decrease of MT3 expression at recurrence in 17/24 tumor pairs (p = 0.002). Conversely, ASPM expression was more frequently positive at relapse (87.5% vs 37.5%, p = 0.03). Loss or deletion of the MT genes cluster was never observed at relapse. Promoter sequencing after bisulfite treatment of DNA from primary tumors and recurrences as well as treatment of short-term ependymoma cells cultures with a demethylating agent showed that methylation was not involved in MT3 downregulation. However, *in vitro* treatment with a histone deacetylase inhibitor or zinc restored MT3 expression.

**Conclusions/Significance:**

The most frequent molecular events associated with ependymoma recurrence were over-expression of kinetochore proteins and down-regulation of metallothioneins. Metallothionein-3 expression is epigenetically controlled and can be restored *in vitro* by histone deacetylase inhibitors.

## Introduction

Ependymomas are tumours thought to derive from radial glial cells [1] and display morphological characteristics of normal ependyma [2]. They represent the third most common intracranial tumour in children and intracranial location account for more than 90% of cases [3]. The incidence is higher in young children as more than fifty percent occur before the age of 5 [4]. The overall prognosis of this tumour remains poor, especially in young children [5] with a 10-year survival between 30 and 70% [6], [7]. Extent of initial surgery remains the only consensual prognostic factor across studies [6], [8], [9]. Recurrences are most of the time local, at least at the beginning of the natural history; distant metastases become more frequent with more effective local treatment [7]. Treatment strategy is actually based on surgery at diagnosis and at each relapse completed with local radiotherapy [4], [6], [10]. The role of chemotherapy is circumscribed to children under 3 years of age to avoid or postpone radiotherapy due to its potential neuropsychological side effects [10], [11]. There is actually no treatment strategy specific for tumour recurrences after radiotherapy.

Advances have been made in our understanding of the molecular mechanisms underlying the oncogenesis of ependymoma with the discovery of specific cancer stem cells [1] and the definition of gene expression profiles specific of each location [1]. In addition, specific molecular signatures associated with clinical characteristics have been identified [12]–[16]. However, reports on prognostic biological markers have shown little consistency or reproducibility [9], [12], [17]–[29]. The Notch pathway, however, has been implicated in three independant studies as a key regulator of ependymoma oncogenesis [1], [12], [30]. Nonetheless, most of these reports concerned only tumours at diagnosis. Progression of ependymoma is possibly related to multiple factors and activated pathways that cannot always be unraveled by tumour analysis at diagnosis.

Aiming at learning more about tumor progression, we hypothesized that relevant information could be obtained by comparing with high throughput technologies tumours from the same patient at diagnosis and at relapse. Dual-color microarray-based gene expression analysis with the two samples labeled with different dyes on the same array, that hybridize competitively to probes on the same spot, allows to adjust for many factors that introduce noise and errors in studies where the comparison of expression differences is made with three different arrays (one for the control and two for the samples at diagnosis and at relapse) [31], [32]. Conversely, this design does not allow to have absolute expression data at diagnosis but only the changes between diagnosis and relapse, but with a higher sensitivity through the limitation of normalization problems [33].

This study revealed pathways specifically up- or down-regulated at relapse that may be used as targets for drug development in pediatric ependymomas. Downregulation of metallothionein-3, also known as neural growth inhibitory factor, was observed at relapse in more than 80% of the recurrences. Conversely, genes of Wnt and Notch pathways were upregulated at recurrence together with numerous genes of the kinetochore and mitotic spindle.

## Materials and Methods

### Tumour material and patient characteristics

Seventeen patients with at least two frozen samples from two different surgeries (one diagnosis and one relapse) were included in this study. Frozen samples of tumour at diagnosis and at least one relapse were obtained for each patient. All samples were snap frozen at the time of surgery. For ten patients, one relapse was available and for seven patients two to three relapses were available. The study encompassed a total of forty-four tumour samples, seventeen at diagnosis and twenty-seven at relapse. Paired tumour samples (diagnosis and relapse) from fourteen patients were obtained from the Tumour Bank at the Necker Enfants Malades Hopital, Paris, France. Two additional paired tumour samples were obtained from the Tumour Bank of the Pierre Wertheimer Hospital, Lyon, France and one from the Neurosurgery Department of the Vrije Universiteit of Amsterdam. The biological study was approved by the Internal Review Board of the Biological Ressource Center of the Necker Sick Children Hospital in Paris, by the Internal Review Board of the Neurosurgery Department of the Vrije Universiteit in Amsterdam and by the Scientific Advisory Board of the NeuroBioTec Tumor Bank in Lyon. Parents/guardians gave their written informed consent for the biological studies performed with the tumor samples.

Patients' characteristics are described in the supplementary data (Table S1). Male to female ratio was 8∶9. Median age at disease onset was 3.4 years (range: 0,4–10,6 years). Tumour location was infratentorial in 11 of 17 patients. Median follow-up of the patients was 42 months (range: 19–96 months). Evaluation of the extent of resection was based on the surgeon's report and post-operative contrast enhanced imaging. External beam irradiation protocol consisted of a local irradiation with surimpression on the operating site. Total radiation doses varied from 50 to 55 Gy and conventional fractioning was used for all irradiated patients. Almost all patients who received chemotherapy were treated according to the BBSFOP protocol [10] except two patients who received fotemustin alone and etoposide alone respectively. When considering the treatment received before a given relapse, we analysed the entire therapeutic sequence between diagnosis and this relapse. Three groups of treatment were considered: surveillance only, chemotherapy only, or irradiation with or without chemotherapy.

Relapse was defined in fifteen patients as a local recurrence of the tumour. In one case, the relapses were loco-regional metastases in the same cerebral hemisphere (Patient 15). In one patient, the relapses were spinal and supra-tentorial metastases of an initially posterior fossa tumour (Patient 3). The median delay between diagnosis and recurrence was 22 months (range: 2.2 – 62.4).

Histological diagnosis and tumour grading review were performed by two independent neuropathologists (PV and FA). Subependymomas and myxopapillary ependymomas were excluded from the study. Before nucleic acid extraction, sections from frozen tumour samples were colored with hematoxylin to discard those containing necrosis or calcifications.

### Nucleic acid isolation

DNA and RNA were extracted from frozen samples with the Microkit (Qiagen). On the forty-four samples studied, eighteen were previously analysed by BAC array-CGH [30]. RNA quality was assessed by 2100 Bioanalyzer® (Agilent Technologies). Quality criteria included 28S/18S ratio >1.2 and RIN (RNA Integrity Number) >8.

### Gene expression array

For each patient, relapses were co-hybridized against their corresponding tumour at diagnosis which served as reference. Probes from tumour tissue and from the reference tissue were differentially labeled by the incorporation of cyanine 3 (Cy3) and cyanine 5 (Cy5) (Dual Color 44K microarray, Agilent Technologies), respectively. Briefly, probes were synthesized from 500 ng of total RNA in two steps according to the manufacturer's instructions. One microgram of purified cRNA from each relapse was mixed with the same amount of diagnosis-tumour cRNA. Hybridizations were performed, in dye-swap, on whole-human-genome 44K oligonucleotide microarrays (product G4112A; Agilent). Feature extraction software provided by Agilent (version 7.2) was used to quantify the intensity of fluorescent images and to apply a Lowess Normalization to correct for artifacts caused by non-linear rates of dye incorporation as well as inconsistencies of the relative fluorescence intensity between some blue and red dyes. All data were imported into Resolver software (Rosetta Biosoftware, Kirkland, WA) for database management, quality control, computational re-combination of dye-swaps, and statistical analysis. Functional analysis was carried out through the Ingenuity Pathway Analysis (Ingenuity® System, http://www.ingenuity.com). Microarray data have been posted on Array Express (IGR_EPENDYMOMA_STUDY_MP ArrayExpress accession number: E-TABM-873, password for reviewer: 1260902888493).

### Comparative Genomic Hybridization (CGH) array

DNA was hybridized to 4×44K whole-genome Agilent arrays (G4426A). For each sample, 500 ng of DNA were fragmented by a double enzymatic digestion (*Alu*I + *Rsa*I) and checked with LabOnChip (2100 Bioanalyzer System, Agilent Technologies) before labeling and hybridization. Tumor DNA and control DNA matched for sex (Promega) were labeled by random priming with Cy5-dCTPs and Cy3-dCTPs, respectively and hybridized at 65°C for 17 h. The chips were scanned on an Agilent G256BA DNA Microarray Scanner and image analysis was done using the Feature-Extraction V9.1.3 software (Agilent Technologies). Feature-Extraction was used for the fluorescence signal acquisition from the scans. Normalization was done using the ranking-mode method, with default value for any parameter. Raw copy number ratio data were transferred to the CGH Analytics v3.4.40 software for further analysis. Raw data have been submitted to the Array Express database (IGR_EPENDYMOMA_CGH_STUDY_MP ArrayExpress accession number: E-TABM-1023, password for reviewer: 1277231149363). The ADM-2 algorithm of CGH Analytics v3.4.40 software was used to identify DNA copy number anomalies at the probe level. A low-level copy number gain was defined as a log2 ratio >0.25 and a copy number loss was defined as a log2 ratio <−0.25. A high-level gain or amplification was defined as a log2 ratio >1.5. DNA copy number anomalies were plotted by the aCGH software package v1.10.0 using the R statistical language.

### Statistical analysis

According to our Gene Expression experimental design, the LogRatios represented the expression changes from diagnosis to recurrence. An initial filtering was applied to retain sequences which appeared as significantly differently expressed (p≤0.01) in at least 50% of recurrences studied. This threshold of 50% was more stringent than the 20% cut-off usually used, but this choice was motivated our decision to include in the statistical analysis only probes which were highly relevant. On this probe set, a one-group t-test was carried out to define a common signature. In this context, the test considered mean (LogR)  =  0 as the null hypothesis. Group comparisons (localization and treatment) were performed using a two-group t-test or an analysis of variance (in case of groups>2) to define differential signatures. For these analyses, the same initial filtering was first applied before carrying out a one-group t-test on each group, independently. This procedure allowed us to retain only probes which were significantly modified in at least one of the compared groups. Finally, the selected probe sets were pooled for the statistical analysis.

For each signature, the networks/pathways search, and functional analysis were generated trough the use of Ingenuity Pathway Analysis®. Briefly, each signature, containing probe identifiers and LogRatio values, were uploaded into the application. Agilent probe identifiers were mapped to their corresponding gene objects in the Ingenuity Pathways Knowledge Base. These genes were then overlaid onto a global molecular network developed from information contained in the Ingenuity Pathways Knowledge Base. Networks of these focus genes were then algorithmically generated based on their connectivity. Identification of biological functions was based on a Fischer's exact test which calculated a p-value determining the probability that each biological function assigned to each signature is due to chance alone.

CGH-array analysis were performed by using the aCGH R package (v1.26.0), and the step down maxT multiple testing procedure of Westfall and Young. Statistical analysis consisted in comparing chromosomal regions imbalances at relapse vs diagnosis, and identifying new abnormalities in recurrences, in general and in association with location or treatment.

### Quantitative Real-Time PCR (qPCR)

Approximately 1 µg of total RNA was used to synthesize cDNA using random hexamers and the Mu-MLV reverse transcriptase (Applied Biosystems). qRT-PCR for the genes MT2A, MT3, KIF11 and ASPM was carried out using Taqman Gene Expression Assays on Demand (Applied Biosystems) and ABI Prism 7700 Sequence Detector (Applera). Expression profile in each specimen was assessed by using the comparative threshold cycle (2^-ddCt^) method. 18S Ribosomal RNA was used as and endogenous control and normal whole brain cDNA (Ambion) as a calibrator.

### Methylation Assay

Investigation of methylation status of the MT3 promoter was assessed by combined bisulfate treatment of genomic DNA and sequencing after PCR amplification. One microgram of genomic DNA was treated with bisulfite, which converts the nonmethylated cytosines to thymines, using the CpGenome™ Universal DNA Modification Kit (Chemicon) according to the manufactor's instructions. PCR amplification was accomplished with primers that do not discriminate between methylated and unmethylated alleles that overlap 4 regions covering the promoter, exon 1 and intron 1 of the MT3 gene, as described [34] and also with two additional pairs of primers (sequences available under request).The PCR products were sequenced using the ABI3730 DNA analyser (Applied Biosystems). The methylation status of CpG islands was determined by direct sequencing of both strands and by estimation of the relative peak height of the PCR products. Normal human DNA and methylated DNA were used as reference control.

### Primary-culture cells

In the absence of an available ependymoma cell line, we used short term cell cultures derived from 2 pediatric ependymomas operated at Necker Sick Childrens Hospital in Paris. Parents/guardians gave written informed consent for research according to the policy of the Internal Review Board of the Biological Ressource Center of Necker Sick Childrens Hospital. Right after surgery, tissues were suspended in DMEM cell culture medium and transferred to the laboratory. After mechanical dissociation, tumour cells were seeded in a 25 cm^2^ flask and maintained in AminioMAX C-100 supplemented medium (Invitrogen) in a tissue culture incubator. Subcultures were processed when cells achieved 80–90% confluence. These primary culture cells were designated as EP1 and EP2. The glial nature of the cultured cells were assessed by morphology and expression of GFAP on immunocytochemistry (Figure S1).

### Epigenetic regulation of metallothioneins expression in vitro

Primary cell cultures of ependymoma and DAOY medulloblastoma cell line (ATCC) were added to 60 mm dish at a density of 5×10^5^ cells and incubated overnight in a 5% CO_2_ incubator. The following day they were treated with 5 µM of 5-Aza-desoxyCytidine (5-Aza), a demethylating agent, for 3 to 7 days (accordingly to the proliferation rate/doubling time of each cell type) or with 300 nM of TSA, a histone-deacétylase inhibitor, for 16 hours. For the combination 5-Aza-dC/TSA treatments, 5-Aza-dC treatment in the same conditions were performed first, followed by identical TSA treatment. Every day, new medium containing freshly prepared 5-Aza was added. At the end of the incubation period, after medium removal, cells were lysed in RTL buffer. RNA was extracted using the RNeasy mini kit (Qiagen) for analysis by qPCR of MT2A and MT3 gene expression levels.

### In vitro regulation of MT3 gene by metal cations and steroids in brain tumor cells Immunohistochemistry

Anti-ASPM affinity purified rabbit polyclonal antibodies were purchased from Bethyl Laboratories Inc (Montgomery, Texas) (reference IHC-00058). Anti-MT3 affinity purified rabbit polyclonal antibody was obtained from Dr Donald Sens (Professor of Pathology, University of North Dakota, School of Medicine and Health Sciences, Grand Forks, ND); their preparation and use on formalin-fixed, paraffin embedded material have been described previously (38, 39). Sections were cut at 4 µm, deparaffinized, exposed to 30 minutes treatment in a steamer at 98°C in citrate pH 7,3 buffer for ASPM and pH 6,0 buffer for MT3 and then treated with a peroxidase blocking agent (reference S2001, DAKO, Glostrup, Denmark). Antibody incubation was performed overnight at 4°C for ASPM (1∶100) and 60 minutes hour at room temperature for MT3 (1∶1000). Antibody binding was visualized with the peroxidase-based anti-rabbit EnVision Kit™ (reference K4003, DAKO) for both antibodies. Diaminobenzidine tetra hydrochloride (DAB, DAKO) was used as chromogen. Sections were counterstained with Mayer's hematoxylin.

Immunohistochemical staining for MT3 was scored semiquantitatively, based on staining intensity and cell number, as follows: 0, no staining; 1, weak staining (independently of the number of positive cells) or staining in less than 10% of cells (independently of the staining intensity); 2, moderate to strong staining in more than 10% of cells. Scoring was performed as of observed in the most positive areas. MT3 generally stained both nucleus and cytoplasm (Figure S2). Staining for ASPM was analysed at high power view (x1000), and scoring was performed as follows: 0, no staining; 1, staining in scarce cells, 2, staining in numerous cells. Following staining patterns were observed for ASPM: cytoplasmic, nuclear, presence of paranuclear “dots” or marked cells in mitosis (Figure S3).

### Tissue micro array

Tissue microarray blocks from ependymoma patients treated with the BBSFOP protocol were built [10]. For each patient, all paraffin blocks and corresponding slides were obtained and reviewed by two neuropathologists (PV, FA) for diagnostic accuracy and tissue adequacy. Sonic aspirator extracts were excluded from the study. Ependymomas were graded based on WHO 2007 criteria. Histopathological findings (ependymal differentiation, necrosis, endothelial proliferation, mitotic index, anaplasia) were evaluated and recorded for each tumour. Immunostainnings for EMA (1∶1, clone E29, DAKO), GFAP (1∶200, clone 6F2, DAKO), OLIG2 (1∶100, RnD systems, Abingdon, UK) and Neurofilament Protein 70 (1∶50, clone 2F11, DAKO) were performed for selected cases. Tumour material was available at diagnosis and at recurrence for 24 patients. There was a total of 29 tumours at recurrence including 17 patients with one recurrence, 6 patients with two recurrences (n = 12) and 1 patient with 3 recurrences (n = 3). Three to four 600 µM-cores were obtained from each tumour. Representative areas were selected whenever present: classical ependymal differentiation (ependymal rosettes, perivascular pseudo-rosettes, and ependymal channels), anaplasia and high vascularisation zones. Normal adult and fetal brain samples were included as internal controls. Frequencies of positivity of MT3 and ASPM at first recurrence were compared to frequencies at diagnosis by McNemar test for paired data, taking into account the intra-patient correlation.

## Results

### Copy number abnormalities with CGHarray

Considering the whole patient population, there was no statistically significant increase in copy number abnormalities from diagnosis to the relapse. The most frequent chromosomal changes between the diagnosis and the relapse were losses of the short arm of chromosome 3 and the long arm of chromosome 6; only the locus 6q25.2 (RBM16, NM_014892) being statistically significant (Figure S4). Copy number changes in 19 regions on chromosome 9 discriminated supratentorial and posterior fossa tumors (Figure S5, Table S2). There was no specific chromosomal copy number variation according to the type of treatment received, albeit loss on chromosomes 3p and 6q were more frequent after radiotherapy (Figure S6).

### Gene expression profiling

We first determined the number of gene expression probe sets differentially expressed between recurrences and initial tumours. These signature volumes were found to be highly variable, ranging from 374 to 18814 probe sets (median: 6275 probes – mean: 9054 probes). The number of probes differentiating the recurrence from its corresponding initial tumour could not be statistically correlated with age at onset, location of the tumour or treatment received but was only linked to the delay between the diagnosis and the relapse. For recurrences occurring before 22 months (ie < to the median delay of recurrence), mean signature included 4799 probes versus 9058 for recurrences that appeared after 22 months (p = 0.013, Student t-test).

**Figure 1:**
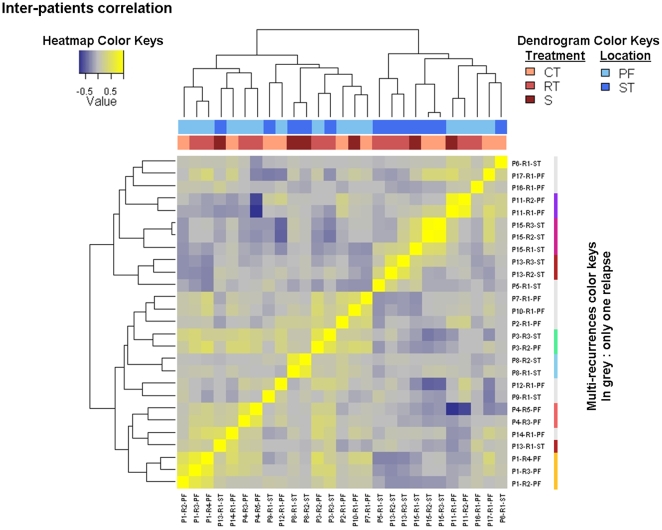
Correlation matrix of the gene expression signatures of the recurrences. After a low-stringent initial filtering (p≤0.01 in at least 20% of experiments), a subset of 29783 probes was used to measure the correlations between relapses (Pearson correlation). As expected, and because each relapse was co-hybridized with its own reference, 6 of the 7 multi-recurrence patients clusterized together. There was however no similar evolution of profiles according to relapse locations or to the treatments received before the recurrence. PF =  posterior fossa, ST =  supratentorial, RT =  radiotherapy, CT =  chemotherapy, S =  surveillance.

To study the molecular signature of the 27 relapses, we used a hierarchical unsupervised clustering for 41000 probes present on the arrays. Recurrences from the same patient were found to be clustered together in 6 out of 7 patients who had experienced several recurrences (Figure 1). Pearson's correlation coefficient between gene expression profiles of recurrences of the same tumour ranged between 0.4191 and 0.8303 (median: 0.5492). As illustrated in the upper lines on Figure 1, localization and adjuvant therapy were not associated with the clustering of recurrences based on their specific expression profile.

### Differences in the recurrence signature according to location

**Figure 2:**
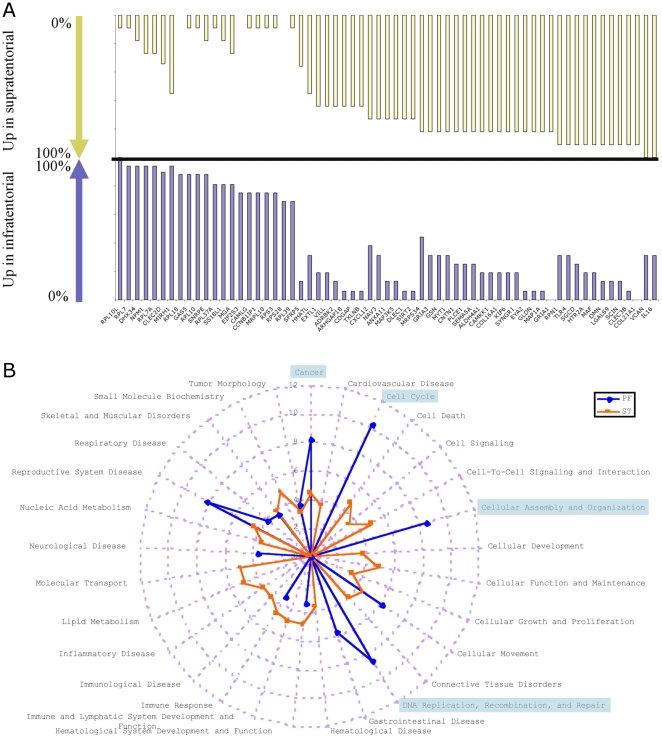
Comparison of functions associated with location of relapses. **A** Gene most frequently upregulated at relapse for supratentorial (upper yellow panel) are represented with the genes most frequently upregulated in infratentorial tumors (lower blue panel). Bars indicated the percentage of tumors in each location with upregulation of the specific gene. **B** The -Log_10_(p-values) of the most discriminatory functions in each group are represented. The p-value for a given function was calculated using the right-tailed Fisher Exact Test by considering 1) the number of uploaded functional analysis molecules that participate in that function, and 2) the total number of molecules that are known to be associated with that function in Ingenuity's knowledge base.

This analysis allowed the identification of 197 genes differentially expressed between the two type of recurrence according to localization (Table S3). A clear difference between frequencies in gene expression according to location was observed. Figure 2A shows the genes most frequently upregulated at recurrence according to the location of the primary tumor. In PF relapses, the ribosomal proteins were the most represented (12 genes). The most abundantly upregulated genes in the relapses of ST ependymomas were involved in cytoskeleton organization (gelsolin, SEMA5A, contactin-1, sarcoglycan, villin-like, scinderin) and extracellular matrix/cellular interactions (gliomedin, EXTL1, galectin-9, desmuslin, tetranectin, versican, COL21A1, COL16A1, CXCL12). A functional analysis of each group signature revealed that the main functional networks associated with posterior fossa relapses were cell cycle, cellular assembly and organization plus DNA Replication, Recombination and Repair (Figure 2B). On the other hand, molecular transport and cell death were evidentiated in the ST relapses.

Although tumor location was not statistically discriminant for the recurrence signature as shown above, our results indicate that progression pattern of supratentorial ependymomas may differ from the one of posterior fossa ependymoma by the overexpression of genes involved in the mesenchymal transition. Conversely, ependymoma recurrences in the posterior fossa progress more often with the overexpression of genes associated with ribosomal functions.

### Differences in the recurrence signature according to treatment

**Figure 3:**
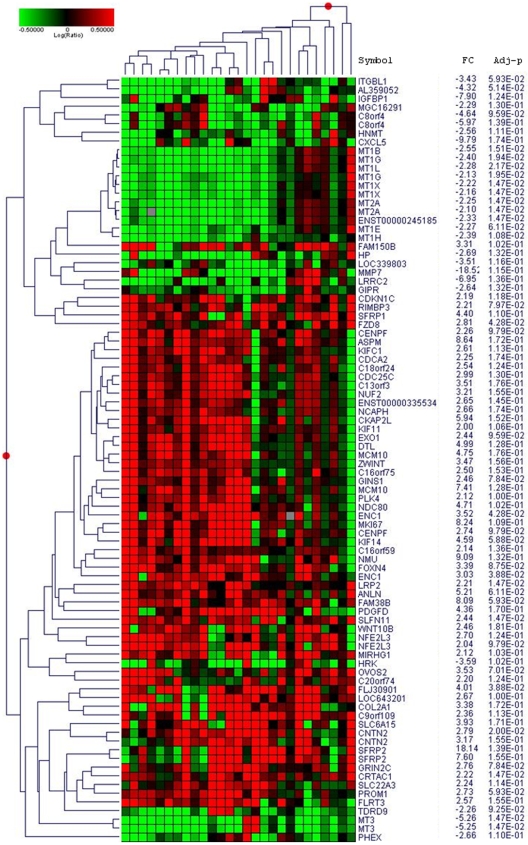
Supervised hierarchical clustering of differentially expressed genes in ependymoma relapse compared to diagnosis. Heatmap showing the 87 genes signature of the genes statistically up- or down-regulated in more than 50% of relapses with a fold change >2. Notice the homogeneity of the underexpression of the metallothioneins.

Specific gene expression profiles of each group (surveillance/chemotherapy only/radiotherapy +/− chemotherapy) (Table S4) failed to identify differentially expressed genes between the chemotherapy and the surveillance group; 58 genes appeared significantly modified in relapses after RT compared to relapses after chemotherapy or surveillance (Figure 3). Recurrences occurring after RT were characterized by downregulation of three potential tumor suppressor genes NKX2-2, YWHAE and WWOX (Student t-test, p<0.01) by at least ten fold and upregulation of HES-2, a known target of NOTCH pathway. Treatment received before recurrence had thus only limited influence on the differential gene expression signature.

### Common signature of recurrences

Considering the limited gene expression signature differences (number of probes and genes) we could identify as influenced by the location of the tumor or by the type of treatment received before the recurrences, we decided to analyse all recurrences together in order to find common genes associated with progression. Considering the poor correlation between the different relapses of the same patient (median Pearson's correlation coefficient  = 0.5492), we decided to include in the analysis all the relapses of patients with multiple relapses.

**Figure 4:**
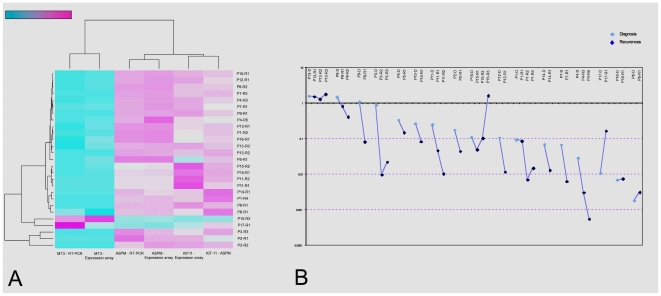
Confirmation analyses (internal validation of gene expression). **A qPCR** Heatmap showing expression of 3 candidate genes (MT3, KIF11, ASPM) in gene expression array and RT-PCR side to side. Pearson correlation coefficients between the two analyses are indicated. **B Evolution of MT3 expression throughout progression.** RT-PCR levels are given as Log scale compared to normal brain.

To identify the specific genes associated with tumour progression, we chose to consider probes with a significant LogRatio (Relapse/diagnosis, p-value≤0.01) in at least 50% of the samples. This filter selected 7384 of the 41 000 initial probes. A one-group t-test was then carried out on this subset of probes by considering LogRatio  = 0 as the null hypothesis. The 298 probes identified were then analyzed in the Ingenuity® database: 240 sequences were mapped, ie related to known genes, 165 were network eligible and 146 were pathway-eligible. This subset of 146 genes was defined as the common signature of ependymoma recurrences (Table S5). A reduced 87-genes signature of specific genes associated with tumour progression is represented in Figure 4 and corresponds to the genes differentially expressed with a fold change ≥2 in at least 50% of the recurrences. This signature is characterized by the activation of the Wnt pathway with overexpression of the following genes SFRP1, SFRP2, FZD2, FZD8, WNT10B besides the upregulation of the stem cell marker CD133 (PROM1) and the proliferation antigen identified by the monoclonal antibody Ki-67 (MKI67). Two other groups of genes were very homogenously differentially expressed in relapses. The first one corresponds to proteins of the kinetochore (KIF14, KIF11, KIF1C, KIF2C, PRC1, BUB1B, ZWINT, ASPM, KNTC2, CENPF), all significantly upregulated. The second one is the group of metallothioneins (MT1L, MT1G, MT1E, MT1X, MT1B, MT2A, MT3) found to be downregulated in 65 to 85% of relapses depending on the MT. MT3, also known as neural growth inhibitory factor, was the most frequently downregulated gene among metallothioneins. The expression of the proliferation marker Ki67 was inversely correlated with MT3 at relapse (Pearson's correlation, r = −0.51, p<0.0001).

Several genes involved in the immune system were found to be downregulated in the common signature of recurrence: CXCL5, CX3CL1, TRAF3IP2, ITGBL1, SERPING1, IFT20, ENTPD3, HP and HPR. Conversely, TIA1, a RNA-binding protein with nucleolytic activity against cytotoxic lymphocytes, was significantly overexpressed (adj. P value  = 0.005).

### Validation of microarray data by qPCR and immunohistochemistry

**Figure 5:**
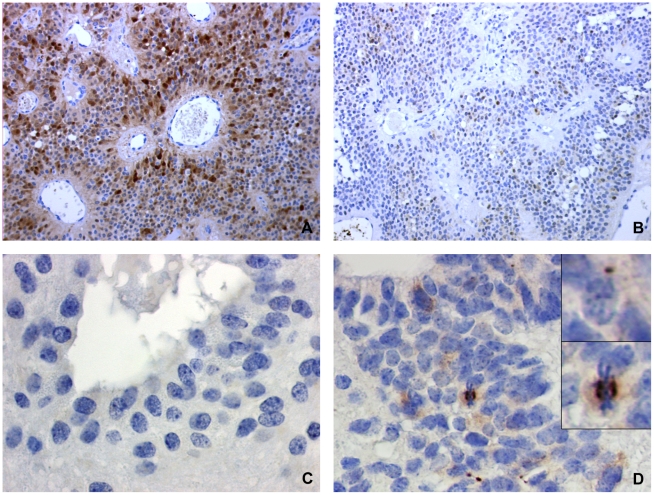
MT3 and ASPM immunostains differ at diagnosis and relapse. (A) Strong nuclear and cytoplasmic staining for MT3 at diagnosis. (B) At relapse the same patient shown at A displayed only weak MT3 staining. Another example of paired tumours, for which ASPM immunostaining was negative at diagnosis (C) and positive at relapse (D). Please also note paranuclear dots and a marked cell in mitosis (D, inserts), two patterns typically observed in ASPM immunostains, together with weak cytoplasmic staining.

Three genes expressed differentially in relapses compared to diagnosis were chosen for further analysis. qPCR analysis were performed for the genes KIF11, ASPM and MT3 in the tumours previously analyzed by gene-expression microarray. The heat-map on Figure 4A illustrates the correlation between microarray and qPCR results. These analyses confirmed the progressive upregulation of the genes KIF11 and ASPM at recurrence. For the MT3 gene, the results showed a low expression level at diagnosis that tended to become even lower throughout relapse. Progressive down-regulation of the MT3 gene expression during progression was thus confirmed for 12 of the 17 (70.5%) patients. Among the 5 patients whose MT3 gene expression was stable or increased during progression, 3 had expression levels below the one of normal brain (Figure 4B). To verify the microarray data at the protein level, immunohistochemistry for ASPM and MT3 was performed on 7 patients among the 17 studied in microarray. The same trend of decreasing MT3 and increasing ASPM staining was confirmed (Figure 5).

### Immunodetection of MT3 and ASPM expression in a independant cohort of pediatric ependymomas

To confirm the changes in expression of MT3 and ASPM in an independent cohort, we studied the expression of these two genes on a TMA of childhood ependymomas composed of 24 tumours at diagnosis with at least one relapse. Among those 24 patients, 23 had a posterior fossa tumour and 1 a supratentorial tumour. Seventeen patients (70.8%) displayed a weaker expression of MT3 at relapse compared to diagnosis (Table 1), 13 of which becoming negative for MT3 during progression. Two patients were negative at both diagnosis and relapse. Four had a stable positive expression of MT3 over all samples, and only one patient had a stronger expression of MT3 at recurrence compared to diagnosis. ASPM staining was stronger at relapse compared to diagnosis in 12 patients (50%), being even negative at diagnosis in ten of then (Table 2). Among the other 12 patients, ASPM staining was identical at diagnosis and relapse, either negative (3 patients) or positive (9 patients). Frequency of positivity was significantly different at diagnosis comparing to relapse for both markers (MT3: p = 0.002 and ASPM: p = 0.03, McNemar test).

**Table 1:** Metallothionein 3 immunohistochemical expression in ependymomas at diagnosis and relapse.

Evolution	Pattern	Patient	D	R1	R2	R3
**Decreased**	++/-	**A**	++	-		
	++/-	**B**	++	-		
	++/-	**C**	++	-		
	++/-	**D**	++	-		
	++/-	**E**	++	-	-	
	++/-	**F**	++	++	-	-
	++/-	**G**	++	+	+	-
	+/-	**H**	+	-		
	+/-	**I**	+	-		
	+/-	**J**	+	-		
	+/-	**K**	+	-		
	+/-	**L**	+	-		
	+/-	**M**	+	+	-	
	++/+	**N**	++	+		
	++/+	**O**	++	+		
	++/+	**P**	++	+		
	++/+	**Q**	++	++	+	
**Stable**	++/++	**R**	++	++		
	++/++	**S**	++	++		
	++/++	**T**	++	++	++	
	+/+	**U**	+	+		
	-/-	**V**	-	-		
	-/-	**W**	-	-	-	
**Increased**	+/++	**X**	+	+	++	

D =  tumor at diagnosis, R1 =  first relapse, R2 =  second relapse, R3 =  third relapse, ++  = medium to strong staining, +  = weak staining, –  =  negative.

**Table 2:** ASPM immunohistochemical expression in ependymomas at diagnosis and relapse.

Evolution	Pattern	Patient	D	R1	R2	R3
**Increased**	-/+	**A**	-	+		
	-/+	**D**	-	+		
	-/+	**S**	-	+		
	-/+	**F**	-	+		
	-/+	**H**	-	+		
	-/+	**E**	-	-	+	
	-/+	**M**	-	-	+	
	-/+	**Q**	-	-	+	
	-/+	**F**	-	-	+	+
	-/+	**G**	-	-	-	+
	+/++	**U**	+	++		
	+/++	**V**	+	++		
**Stable**	-/-	**B**	-	-		
	-/-	**C**	-	-		
	-/-	**I**	-	-	-	
	+/+	**L**	+	+		
	+/+	**N**	+	+		
	+/+	**O**	+	+		
	+/+	**P**	+	+		
	+/+	**K**	+	+		
	+/+	**R**	+	+		
	+/+	**S**	+	+		
	+/+	**W**	+	+	+	
	+/+	**T**	+	+	+	

D =  tumor at diagnosis, R1 =  first relapse, R2 =  second relapse, R3 =  third relapse, ++  = medium to strong staining, +  = weak staining, –  =  negative.

### Mechanism of regulation of metallothionein in ependymomas

Metallothioneins being the most homogeneously downregulated genes at relapse, we decided to investigate the possible mechanisms of their repression at the genetic and epigenetic levels.

Considering that all MT genes are clustered on chromosome 16q13, we first verified a possible deletion of this chromosome region. CGH array analysis did not show a loss for this chromosome region at relapse (Figure S5). To rule out the possibility of a small genomic deletion missed by CGHarray analysis we carried out quantitative PCR analysis for the MT2A gene. Amplification products could be obtained in all 37 samples tested with a CT corresponding to the one of normal DNA reference (Figure S7).

The absence of DNA deletion prompted us to investigate the regulation of gene expression at transcriptional level. We first verified whether genes known to interact with the MT gene promoters were differentially expressed at relapse. None of the transcriptional activators (MTF1, USF1, NF1, STAT3, IL6) was found to be down-regulated at relapse compared to diagnosis. None of the transcriptional repressors (SIN3A, SIN3B, MTA1, HDAC1) was found to be upregulated.

**Figure 6:**
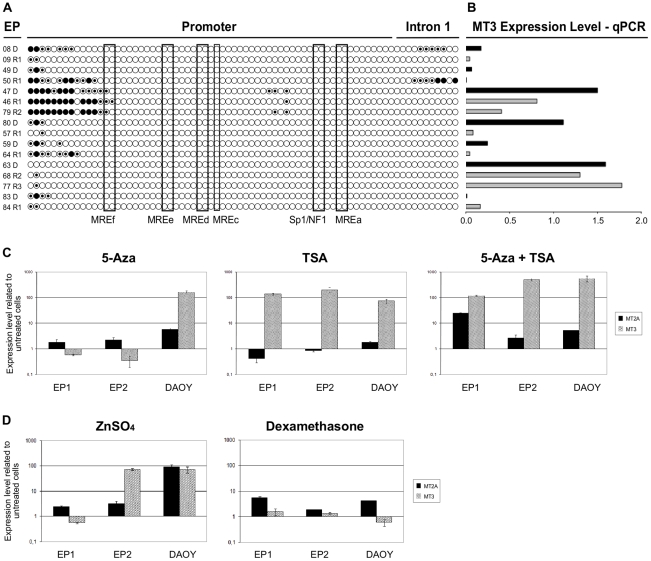
A: Methylation status of 74 CpG sites at MT3 promoter and intron 1. Each row of circles represents one EP sample sequenced from PCR products generated from amplification of bisulfite-treated DNA. Empty circles  =  unmethylated cytosines; Dotted circles  =  hemimethylated cytosines; Dark circles  =  methylated citosines. **B: MT3 expression analysis by quantitative PCR in the corresponding ependymoma tumors.** Samples with results under 1.0 are downregulated and those over 1.0 are upregulated compared to normal brain. The black bars correspond to sample at diagnosis, and the grey bar to relapse. Each histogram represent the corresponding sample studied for methylation. **C: Epigenetic modulation of MT2A and MT3 expression** on short term ependymoma cultures EP1 and EP2 and medulloblastoma cell line DAOY as control. Demethylation by 5-Aza-Deoxycytidine (left panel). Histone deacetylation inhibition by Trichostatin A (middle panel). Combined treatments (right panel). **D: Treatment with zinc sulfate restores the expression of MT3.** MT2A and MT3 expression level after 24 hours of 200 mM of ZnSO4 (left panel) and 5 microM of dexamethasone (right panel) treatments in the ependymoma primary culture cells EP1 and EP2 and in the medulloblastoma cell line DAOY.

Since differential expression of regulatory factors could not explain MT3 downregulation, we investigated whether epigenetic factors and especially CpG islands methylation, were implicated in MT3 down-regulation at relapse. If methylation would be the cause of MT3 downregulation, more methylated CpG islands should be observed at relapse. We observed limited to no methylation of the 74 CpG islands in regulatory regions and intron 1 of MT3 and no increasing methylation at relapse (Figure 6A). None of the few methylated sites was correlated with gene expression measured by qPCR (Figure 6B). To confirm this data, ependymoma primary cells (EP1 and EP2) were treated with the demethylating agent 5-Aza-DeoxyCytidine (5-Aza) followed by MT3 and MT2Agene expression analysis by qPCR (Figure 6C). DAOY, a medulloblastoma cell line was used for comparison. The 5-Aza treatment alone induced a small increase in MT2A expression level in all cells tested (1.8, 2.2 and 5.7 fold for EP1, EP2 and DAOY respectively). The 5-Aza treatment alone did not increase MT3 expression in the ependymoma cells, while a 2 log increase in expression was found for DAOY. This data confirms the CpG islands methylation results, since the EP1 and EP2 exhibit very few methylated sites on the contrary to DAOY cells which harbor a hypermethylated pattern on MT3 promoter region (data not shown).

To test if epigenetic inactivation of MT genes could be due to histone acetylation, cells were also treated with trichostatin A (TSA), a deacethylating agent, alone or in combination with 5-Aza (Figure 6D). On the other hand, TSA proved effective in increasing dramatically MT3 expression in all tested cells: 135 fold for EP1, 198 fold for EP2, and 73 fold in the DAOY. MT2A expression level did not change significantly in the ependymoma primary and in DAOY cells after TSA treatment. Combination of 5-Aza and TSA was more effective than either agent alone in increasing MT3 levels in EP2 and in DAOY cell lines. MT2A levels in EP cells were significantly increased after a treatment combining 5-Aza and TSA compared to either treatment alone.

Epigenetic modulation of EP cells *in vitro* confirmed that MT3 expression was not regulated by promoter methylation but more likely by histone acetylation status. On the contrary, MT2A was not regulated only by histone acetylation status but also by methylation.

The MT3 promoter presents many regulatory elements, such us MRE (Metal Responsive Element) that allows MT induction by metal cations through MTF1 transcription factor and GRE (Glucocorticoid Responsive Element) for glucocorticoids induction. To check chromatin accessibility, we treated ependymoma primary cells EP1 and EP2 and DAOY cell line with ZnSO_4_ and dexamethasone (Figure 6D). Both agents were able to induce MT2A expression in all cells tested. Nevertheless MT3 was induced by ZnSO_4_ in only one of the two EP cells and in DAOY. Dexamethasone did not induce a significant upregulation of MT3 in any of the three cell lines tested. These results suggest that the MRE in the promoter of MT3 is not always accessible in EP cells *in vitro*.

## Discussion

Despite several molecular studies, the oncogenesis of ependymoma remains elusive. Specific molecular events occurring during progression have only been seldom reported [30], [35]. This work focused on recurrence-specific gene expression signature variations. We choose a dual color microarray system in order to maximize the likelihood to discover significant changes in gene expression. The tumour of the patient at diagnosis was used as the reference and marked with Cy5 and the tumour of the same patient was marked with Cy3 and co-hybridized competitively. Consequently, only real changes in gene expression occurring at relapse were detected. As ependymoma's cell of origin remains uncertain, we also thought that most of the gene expression studies have suffered from the lack of specificity of the reference mRNA used. When comparing the tumour with the normal brain, most of the genes that are overexpressed correspond to cellular processes linked to proliferation while most of the genes downregulated correspond to neuronal proteins. Moreover, it has been recently assumed that glial tumours display brain-region specific expression profiles regardless of the tumour histology [1], [12], [14], [36]. To analyse properly gene expression changes compared to the control, one should therefore use the normal brain control from the same location as the tumor. The brain region-specific expression pattern should remain stable over time between diagnosis and local recurrence. It is thus not surprising that none of the genes described as location–specific at diagnosis appeared in our recurrence signature.

Microarray studies conducted to discover molecular pathways linked with tumour progression and papers comparing metastases to initial tumours have already been performed [37]–[41]. The use of paired samples alleviate the bias associated with interindividual variation; for example, comparison of expression profile of tumours prior to and following systemic chemotherapy allowed the identification of differentially expressed genes correlated with chemoresistance in ovarian carcinomas. [37] In addition, paired samples by reducing variability increase strikingly the statistical power of the study [42].

Microarray analysis of recurrence-specific expression changes demonstrates the existence of a common signature for recurrence in ependymoma. This signature pinpoints pathways already described in other ependymoma studies focusing on tumours at diagnosis such as the Wnt and the Notch pathways [12], [14], [30]. The common signature at relapse also unveils several genes already described in the death-from-cancer signature [43] including MKI67, KNTC2 (HEC1) and BUB1B. These last two genes belong to a broader group of molecules overexpressed at relapse in ependymoma and playing a role in spindle formation. Several kinetochore molecules were found in our signature and have already been described as prognostic markers in other tumours: KNTC2 in lung cancer and kinesin KIF14 in breast and lung cancer [44]–[46]. The spindle molecule ASPM has been shown to be involved in the malignant progression of gliomas possibly through expansion of a cancer stem cell compartment [47]. Beside their prognostic value, these molecules may also represent new therapeutic targets. Classic spindle poisons target tubulin and have not demonstrated their efficacy in ependymoma. But new chemotherapies, known as kinesin spindle inhibitors, are actually under evaluation. Among them, monastrol, a kinesin Eg5 (KIF11) inhibitor, has already demonstrated its efficiency in glioma in vitro [48]–[50] and is currently under clinical development.

Apart from upregulation of genes associated with proliferation, the key event associated with recurrence in our common signature was down-regulation of metallothioneins, especially MT3. The metallothioneins are small proteins that posses about 60 amino acids, with a high level of cysteines that confers to then the ability to bind divalent metals. Metallothioneins function as metals reservoirs, maintaining metal homeostasis and contributing to heavy metals detoxification, phenomenon that can lead to chemoresistance in some cancers, [51]–[53] and scavenging free radicals [54]. In mammals there are four groups of MT proteins: MT1, MT2, MT3 and MT4, that are coded by a family of genes clustered on chromosome 16q13. MT2 protein coded by MT2A gene accounts for 80% of the MTs proteins. The MT1 and MT2 are ubiquitously expressed. MT3 was first detected in the brain of patients with Alzheimer's disease, identified as a factor inhibiting neuronal growth in culture and called neural GIF (growth inhibitory factor) [55]. MT3 is expressed predominantly within the CNS and has been found both in neurons and in astrocytes [56]. MT3 is expressed at a lower level in other tissues such as kidney [57], [58]. MT4 is specifically expressed in the stratified squamous epithelium [59]. A number of studies have shown enhanced synthesis of MTs in proliferating tissues suggesting its crucial role in normal and neoplasic cell growth [60] but their precise role in carcinogenesis is still unclear, once they can also act as oncosupressor [61]. In several carcinomas indeed, metallothioneins are downregulated compared to the tissue of origin [34], [62]–[65]. In ependymoma, the expression of MT 1-2 has been studied at diagnosis by immunohistochemistry; MT1-2 positivity was statistically more frequent in low grade ependymomas and was associated with a better survival [66]. To the best of our knowledge, our study is the first one focusing on MT3 and displaying immunostains for MT3 in brain tumors. Of note, in our controls consisting of normal adult and fetal brain we observed prominent immunostaining for MT3 in astrocytes but no staining in neurons (Figure S2). Since no genomic loss was observed on the chromosome 16q13 region in our ependymoma samples, this downregulation was more likely to be linked to transcriptional inactivation.

Former descriptions of MT3 inhibition by promoter methylation [34] prompted us to perform a methylation assay on the MT3 gene in ependymomas. No significant hypermethylation was observed, even if we consider exclusively the intron 1, reported to be the region abnormally hypermethylated associated with low MT3 expression in gastric carcinoma cells [44]. The inability of 5-Aza to restore MT3 expression in EP cells confirmed these data.

Since all metallothioneins were homogeneously downregulated at recurrence compared to diagnosis, we hypothesized that chromatin changes in the 16q13 region of the MT genes cluster could explain their repression. Histone deacetylases can regulate expression of tumor suppressor genes and activities of transcriptional factors involved in both cancer initiation and progression through alteration of either DNA or the structural components of chromatin. We therefore used the prototypic histone deacetylase inhibitor TSA to modulate MT expression. While MT3 expression was restored by TSA treatment, this was not the case for MT2A, shown above to be also dependant on methylation. The TSA effect could be explained either by inhibition of HDAC1, a known repressor of MT genes or by opening of the chromatin structure and upregulation of MTF1[67]. Although the main regulatory event for MT3 seems to be associated with histone acetylation, the synergistic effect observed in EP2 by combining TSA and 5-Aza suggest that other methylated genes or histones maybe indirectly involved in the regulation of MT3. Histone deacetylase inhibitors may therefore be interesting drugs in ependymomas.

While the expression of MT3 and MT4 are constitutive and tissue-specific, MT1 and MT2 expressions are more ubiquitous and highly inducible by a variety of developmental and environmental signals, such as metals, oxidative stress, cytokines, glucocorticoids hormones and irradiation [68]. In this work, we show the possibility to induce MT3 expression with zinc in brain tumor (EP and medulloblastoma) cells. However, metal-responsive element in the promoter of MT3 are not accessible in all EP cells. Indeed, MT3 has been considered for a long time as a non metal-inducible gene in normal astrocytes and neurons cultures [69], [70], but recently Wei and co-workers showed MT3 induction after zinc treatment in prostate cancer cells [71]. Due to poor penetration of zinc into the brain, modulating MT3 expression in ependymomas with this cation would need proper formulations.

Several genes involved in the immune system were found to be downregulated in the common signature of recurrence (Figure 2B, Table S5); some of them being already reported by Donson et al, as associated with the absence of recurrence [72]. Our data are thus consistant with the hypothesis of these authors suggesting a role for the immune system to prevent recurrence in ependymoma.

Analysis of gene expression profiles specific of each location pointed out overexpression of genes related to the epithelial-mesenchymal transition in supratentorial locations [72]. The overexpression of genes involved in cytoskeleton organization as well as those involved in cel/cell and cell/matrix interactions could explain the higher invasive capacities of these tumors at the time of relapse. Contactin 1 (CNTN1), for example, has already been proposed as a key factor in glioma dissemination and its expression tends to be increased in several brain tumours [73]. In addition, contactin 1 has recognized interactions with developmental control genes belonging to the Notch pathway [74]. With respect to the recurrences of posterior fossa ependymomas, the upregulation of ribosomal proteins is consistent with increased proliferation usually seen in these tumors at recurrence, depicted for example by increased Ki67 labeling; in medulloblastomas as well, the overexpression of ribosomal proteins has been shown to be the hallmark of aggressive tumors[75].

The analysis of genes specifically downregulated at relapse after radiotherapy identified NKX2-2, a transcription factor involved in glioma histogenesis [76]. Its repression is associated with the blockade of oligodendrocyte differentiation [77] and the oncogenic phenotype of cancer [76].

### Conclusion

Our data suggests that the gene expression profile of ependymoma shows limited but significant changes upon relapses. This gene expression profile is only minimally influenced by the treatments used. However, the changes in expression profile at recurrence were linked to some extent with the location of the initial tumor. Despite interindividual variations, ependymoma relapses display a common gene expression signature that is marked by the upregulation of kinetochore proteins and downregulation of metallothioneins. The therapeutic strategies targeting kinesin proteins or those aiming at restoring metallothionein expression, such as histone deacetylase inhibitors, deserve further study in these tumours.

## Supporting Information

Table S1 Clinical characteristics of the patients. D  =  diagnosis. R  =  recurrence. Delay of relapse in days. BBSFOP  =  polychemotherapy protocol, see ref 7. RT  =  radiotherapy. PF  =  posterior fossa, ST  =  supratentorial, SPI  =  spinal. DOD  =  dead of disease, ADF  =  alive disease-free, AWD  =  alive with disease. Time of follow-up in months.
(0.03 MB XLS)

Table S2 Chromosome 9 imbalances differentiating posterior fossa and supratentorial ependymomas. Corresponding genes are indicated in the second column.
(0.18 MB XLS)

Table S3 List of genes differentially regulated according to location of the initial tumor. The site of the relapse was not identical to the initial site in only one case (Pt3).
(0.16 MB XLS)

Table S4 List of genes differentially regulated according to the treatment(s) received. All treatments received between diagnosis and relapse are considered. Patients having received chemotherapy and radiotherapy between diagnosis and the relapse considered are included in the radiotherapy group for the analysis.
(0.21 MB XLS)

Table S5 List of genes differentially expressed at recurrence.
(0.07 MB XLS)

Figure S1 Ependymoma short-term cultures. Cells were cultured after mechanical dissociation of fresh tumor material kept in DMEM. Low passages (5th to 15th) were used for the experiments.
(1.32 MB DOC)

Figure S2 Metallothionein 3 (MT3) staining of normal brain. MT3 is detected in the astrocytes but not in the neurons (panel A) nor in the oligodendrocytes (panel B).
(3.97 MB TIF)

Figure S3 ASPM staining of ependymomas. ASPM is detected in the mitotic spindle in every phase of the mitosis, as well as in the cytoplasm of cells not in mitosis.
(10.17 MB TIF)

Figure S4 Comparison of CGHarray profiles at diagnosis and at relapse. D  =  diagnosis; R  =  relapse.
(0.22 MB TIF)

Figure S5 Chromosomal imbalances that are distinct in supratentorial and posterior fossa tumors. ST  =  supratentorial; PF  =  posterior fossa.
(0.20 MB TIF)

Figure S6 Chromosomal changes at relapse according to treatment received. CT  =  chemotherapy; RT  =  radiotherapy; Surv  =  surveillance.
(0.16 MB TIF)

Figure S7 Quantitative PCR of MT2A gene in ependymoma samples.
(0.03 MB DOC)
