# Supplementary material for: Portrait of Ependymoma Recurrence in Children: Biomarkers of Tumor Progression Identified by Dual-Color Microarray-Based Gene Expression Analysis
Source: PLoS One. 2010 Sep 24;5(9):e12932. doi: 10.1371/journal.pone.0012932 (PMC2945762; doi:10.1371/journal.pone.0012932)
Supplement: Figure S1 — Ependymoma short-term cultures. Cells were cultured after mechanical dissociation of fresh tumor material kept in DMEM. Low passages (5th to 15th) were used for the experiments. (1.32 MB DOC) [file pone.0012932.s006.doc]

# Figure S1 : EPENDYMOMA SHORT-TERM CULTURES


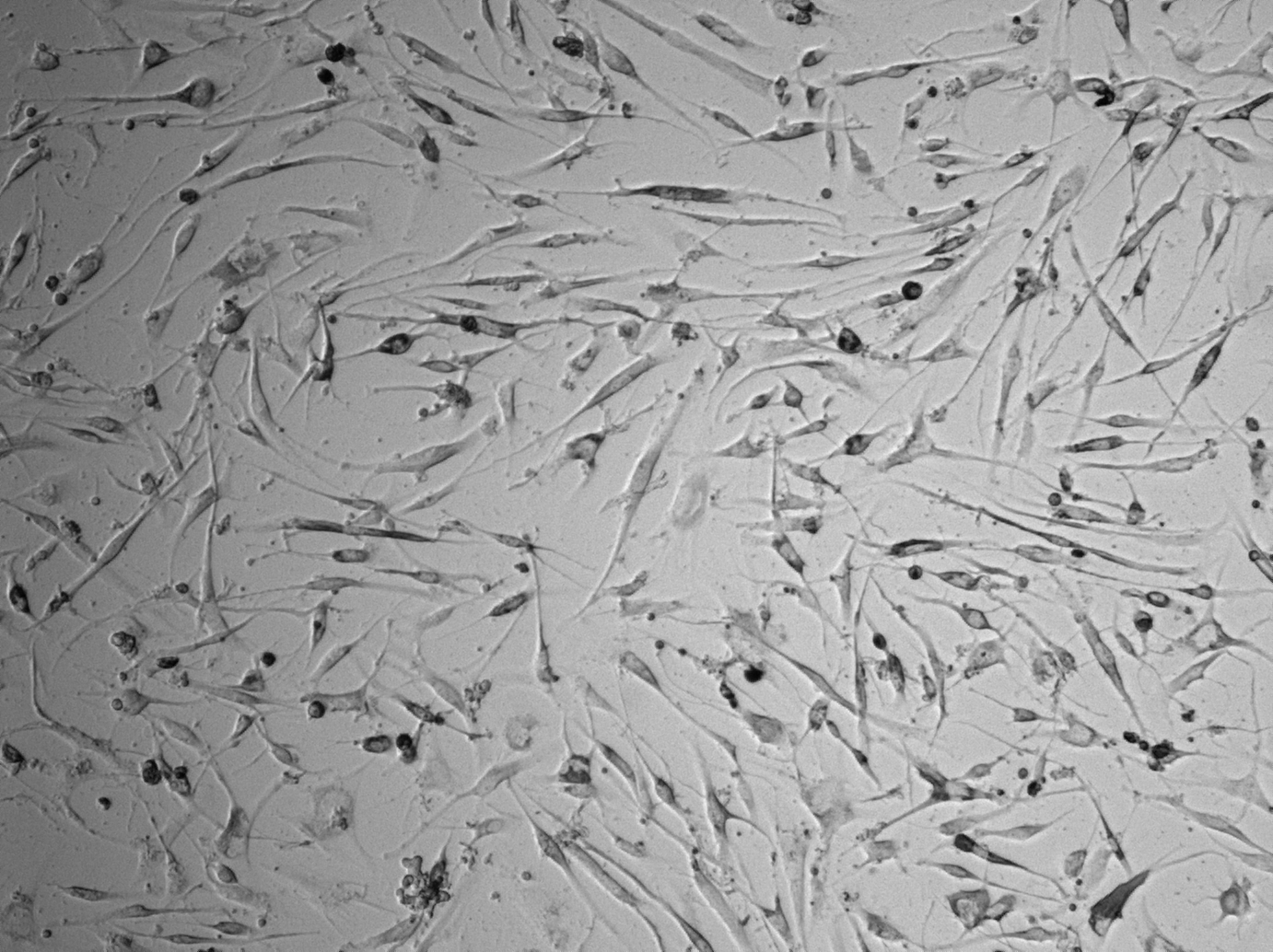


EP1 : from a posterior fossa tumor in a 6y old girl


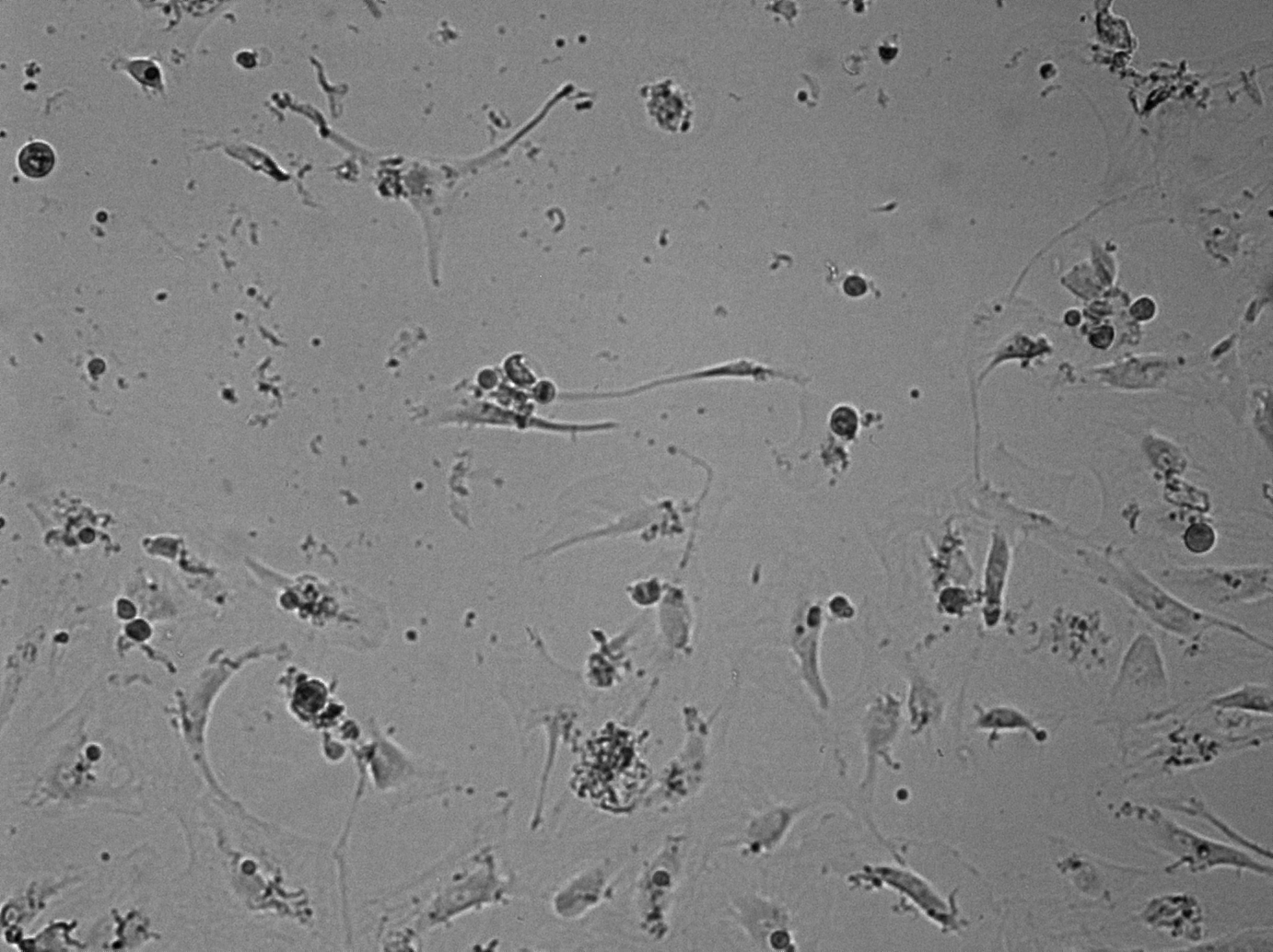


EP2 : from a supratentorial ependymoma in a 14y old girl

|  | Immunohistochemistry | | | | | |
| --- | --- | --- | --- | --- | --- | --- |
| Cell line | GFAP | PS100 | Synaptophysin | NEUN | NSE | EMA |
| EP1 | 1(focal) | 0 | 0 | Not done | 3 | 0 |
| EP2 | 3 | 3 | 1 | Not done | 3 | 0 |
