# Supplementary figures and images for: Portrait of Ependymoma Recurrence in Children: Biomarkers of Tumor Progression Identified by Dual-Color Microarray-Based Gene Expression Analysis
Source: PLoS One. 2010 Sep 24;5(9):e12932. doi: 10.1371/journal.pone.0012932 (PMC2945762; doi:10.1371/journal.pone.0012932)

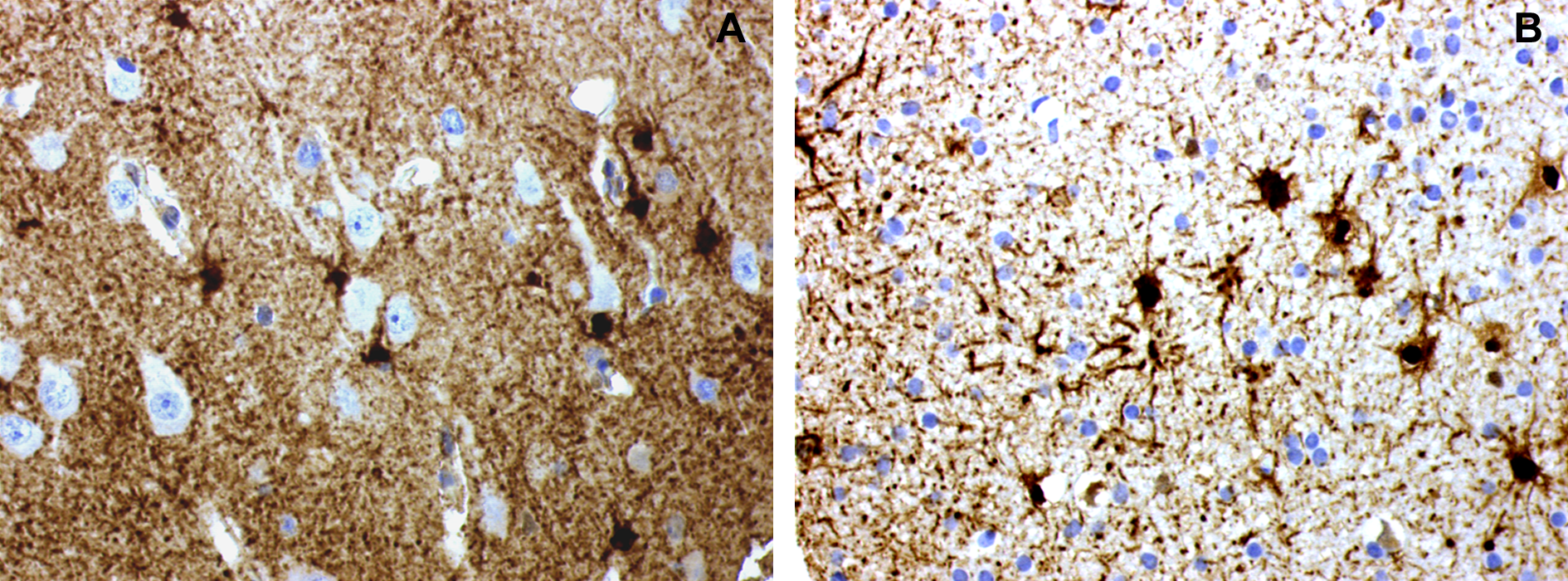

Supplement: Figure S2 — Metallothionein 3 (MT3) staining of normal brain. MT3 is detected in the astrocytes but not in the neurons (panel A) nor in the oligodendrocytes (panel B). (3.97 MB TIF) [file pone.0012932.s007.tif]

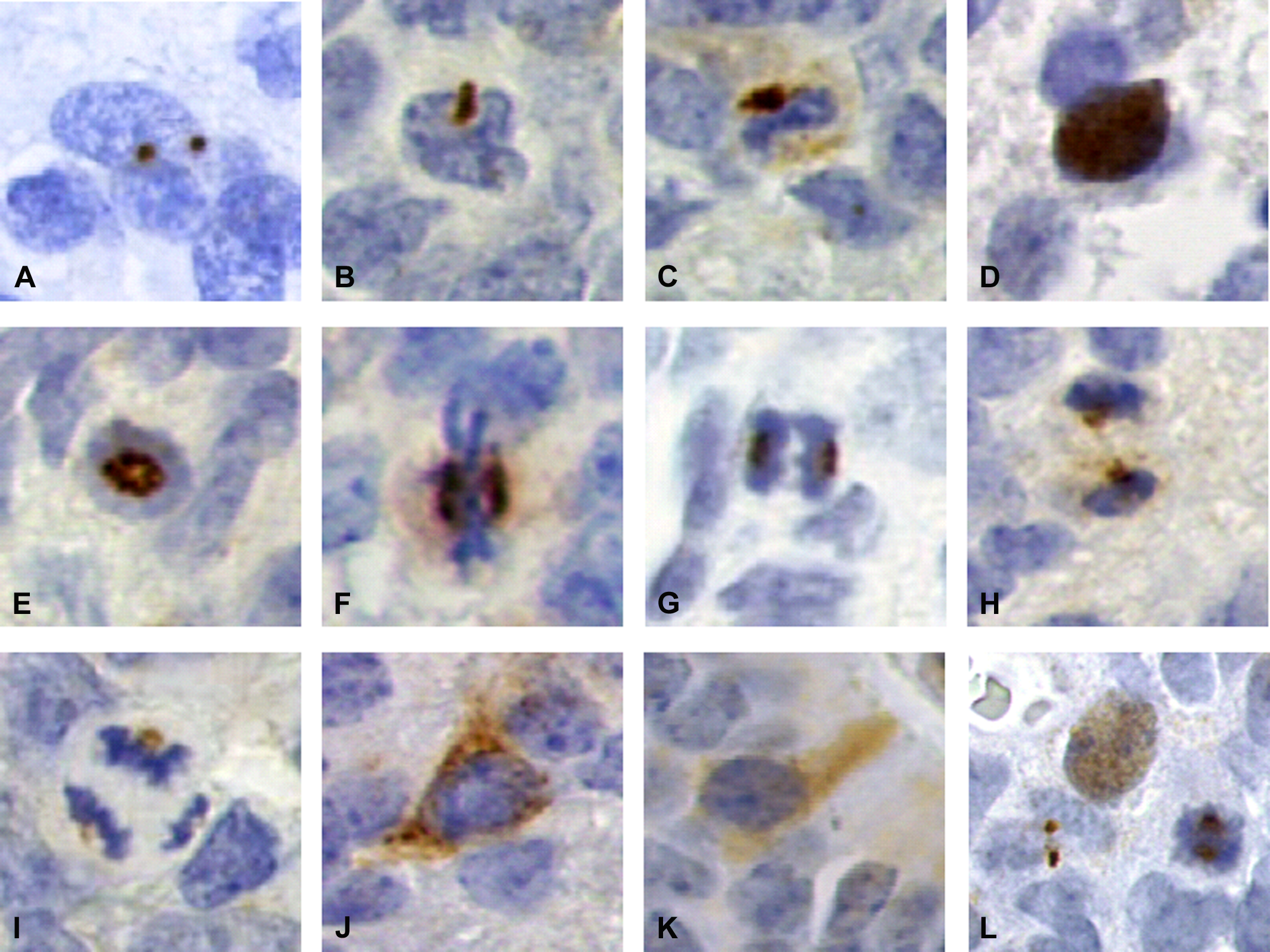

Supplement: Figure S3 — ASPM staining of ependymomas. ASPM is detected in the mitotic spindle in every phase of the mitosis, as well as in the cytoplasm of cells not in mitosis. (10.17 MB TIF) [file pone.0012932.s008.tif]

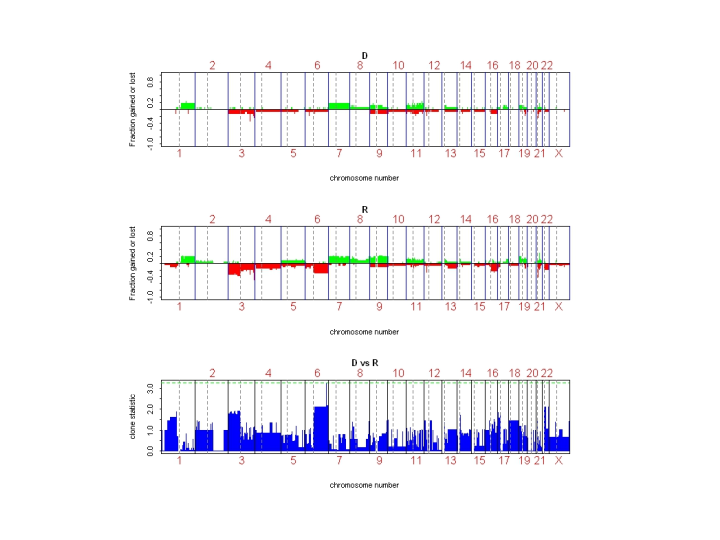

Supplement: Figure S4 — Comparison of CGHarray profiles at diagnosis and at relapse. D = diagnosis; R = relapse. (0.22 MB TIF) [file pone.0012932.s009.tif]

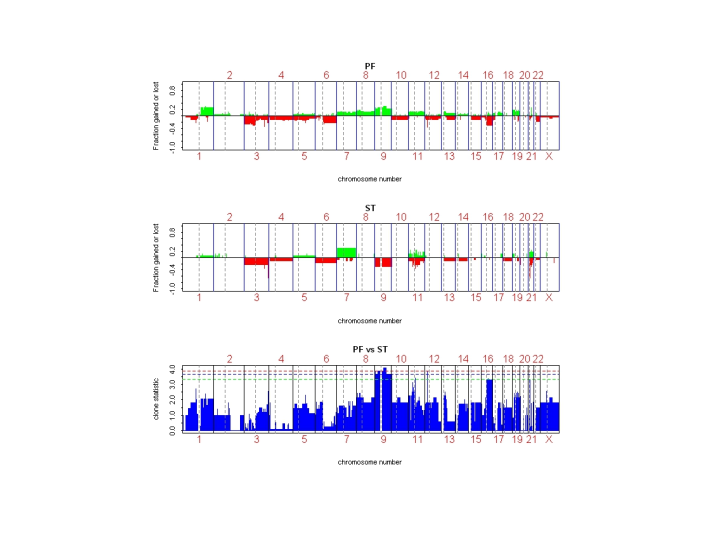

Supplement: Figure S5 — Chromosomal imbalances that are distinct in supratentorial and posterior fossa tumors. ST = supratentorial; PF = posterior fossa. (0.20 MB TIF) [file pone.0012932.s010.tif]

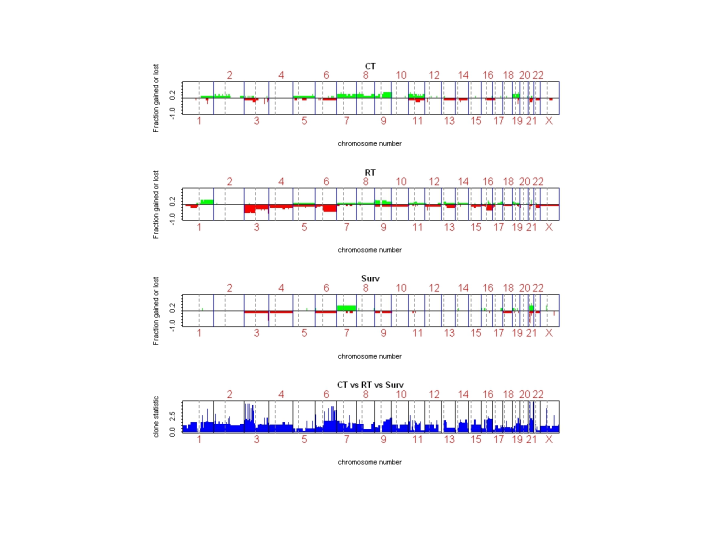

Supplement: Figure S6 — Chromosomal changes at relapse according to treatment received. CT = chemotherapy; RT = radiotherapy; Surv = surveillance. (0.16 MB TIF) [file pone.0012932.s011.tif]
