# Supplementary material for: Portrait of Ependymoma Recurrence in Children: Biomarkers of Tumor Progression Identified by Dual-Color Microarray-Based Gene Expression Analysis
Source: PLoS One. 2010 Sep 24;5(9):e12932. doi: 10.1371/journal.pone.0012932 (PMC2945762; doi:10.1371/journal.pone.0012932)
Supplement: Figure S7 — Quantitative PCR of MT2A gene in ependymoma samples. (0.03 MB DOC) [file pone.0012932.s012.doc]

Supplementary data

Figure S7: qPCR analysis in EP samples. 10 ng of DNA was used in each PCR. The CT values are represented. Ependymoma samples and DNA reference used as a control. The obtained PCR product indicates that no DNA deletion is observed in the samples.
